# Galectin-4 Reduces Migration and Metastasis Formation of Pancreatic Cancer Cells

**DOI:** 10.1371/journal.pone.0065957

**Published:** 2013-06-18

**Authors:** Ana I. Belo, Astrid M. van der Sar, Boris Tefsen, Irma van Die

**Affiliations:** 1 Department of Molecular Cell Biology and Immunology, VU University Medical Centre, Amsterdam, The Netherlands; 2 Department of Medical Microbiology and Infection control, VU University Medical Centre, Amsterdam, The Netherlands; Faculdade de Medicina, Universidade de São Paulo, Brazil

## Abstract

Galectin-4 (Gal-4) is a member of the galectin family of glycan binding proteins that shows a significantly higher expression in cystic tumors of the human pancreas and in pancreatic adenocarcinomas compared to normal pancreas. However, the putative function of Gal-4 in tumor progression of pancreatic cancer is still incompletely understood. In this study the role of Gal-4 in cancer progression was investigated, using a set of defined pancreatic cancer cell lines, Pa-Tu-8988S (PaTu-S) and Pa-Tu-8988T (PaTu-T), as a model. These two cell lines are derived from the same liver metastasis of a human primary pancreatic adenocarcinoma, but differ in their growth characteristics and metastatic capacity. We demonstrated that Gal-4 expression is high in PaTu-S, which shows poor migratory properties, whereas much lower Gal-4 levels are observed in the highly metastatic cell line PaTu-T. In PaTu-S, Gal-4 is found in the cytoplasm, but it is also secreted and accumulates at the membrane at sites of contact with neighboring cells. Moreover, we show that Gal-4 inhibits metastasis formation by delaying migration of pancreatic cancer cells *in vitro* using a scratch assay, and *in vivo* using zebrafish (*Danio rerio*) as an experimental model. Our data suggest that Gal-4 may act at the cell-surface of PaTu-S as an adhesion molecule to prevent release of the tumor cells, but has in addition a cytosolic function by inhibiting migration via a yet unknown mechanism.

## Introduction

Pancreatic cancer is one of the leading causes of death by cancer in the western world. Clinically efficient strategies for early detection of the disease are still not available. Incidence of pancreatic cancer and mortality in patients with this type of cancer has hardly decreased over the last 50 years [1], [2], [3], [4]. Therefore further understanding in the mechanisms of onset, progression and metastasis of pancreatic cancer is warranted.

Galectins are proteins that can be aberrantly expressed in cancer and have been implicated in cancer progression [5], [6]. They consist of a family of galactoside-binding soluble lectins that have been classified into three subgroups based on their structure and number of carbohydrate-recognition domains: prototype (galectins-1, -2, -5, -7, -10, -11, -13, and -14), chimera type (galectin-3), and tandem repeat type (galectins-4, -6, -8, -9, and -12) [reviewed in [7]]. They function in a wide variety of biological processes both intra- and extracellularly. Galectin–glycoprotein lattices play major roles in the regulation of cell function, like cell–cell adhesion, cell–extracellular matrix (ECM) interactions, cell growth [8], organization of membrane domains in lipid raft formation [9], [10], [11], leucocyte migration [reviewed by [12]] and regulation of intracellular signaling [13], [14], [15].

The expression of galectins is modulated during the development of individual cells and can be altered under different physiological or pathological conditions. Galectins are often overexpressed in cancerous cells and cancer-associated stromal cells, especially in those cell types that normally do not express the specific galectin [16]. In cancer progression, galectins are involved in differentiation, adhesion, migration, angiogenesis, malignant transformation, apoptosis and cancer drug resistance [reviewed in [5], [17], [18], [19], [20], [21], [22]]. Furthermore, there are several reports that have linked these proteins to invasion and metastasis in several types of cancers [16], [23], [24], [25], [26], [27], [28].

In this study we have focused on the role of galectin-4 (Gal-4) in metastasis of pancreatic cancer cells. Metastasis formation is a multi-step process in which primary tumor cells invade neighboring tissues, migrate through the vasculature to finally extravasate into the perivascular tissue and proliferate into secondary tumors. Gal-4 is a 323-amino acid (36 kDa) protein that is predominantly expressed in the luminal epithelia of the gastrointestinal tract, from the tongue to the large intestine. Gal-4 expression is not detected in healthy pancreas, but is significantly enhanced in cystic tumors of the human pancreas and pancreatic adenocarcinomas compared to normal tissue samples, whereas its expression is low in pancreatic neuroendocrine tumors [26], [27], [29], [30], [31]. The function of Gal-4 in tumor progression and metastasis in pancreatic cancer, however, remains unclear. In this study the putative role of Gal-4 in cancer progression was investigated, using a set of defined pancreatic cell lines. The results demonstrate that Gal-4 is higher expressed in the more differentiated pancreatic tumor cells compared to pancreatic cells demonstrating metastatic capabilities. Moreover, Gal-4 affects metastasis formation by delaying migration and metastasis of pancreatic cancer cells *in vitro* in a scratch assay and *in vivo* in zebrafish embryos as an experimental model.

## Materials and Methods

### Ethics Statement

Roy^−/−^;nacre^−/−^ casper *Danio rerio* (zebrafish) were handled in compliance with the local animal welfare regulations and maintained according to standard protocols (zfin.org). The breeding of adult fish was approved by the local animal welfare committee (Animal Experimental licencing Committee, DEC) of the VU University medical center. All protocols adhered to the international guidelines specified by the EU Animal Protection Directive 86/609/EEC, which allows zebrafish embryos to be used up to the moment of free-living (approximately 5–7 days after fertilisation). Because embryos used in this study met these criteria, no DEC licence is required for this study.

### Antibodies, Reagents and Buffers

Goat anti-human Galectin-4 (BD Biosciences, Belgium) was used for detection of Gal-4 and mouse anti-tubulin (Cedarlane, Canada) was used as an endogenous control. Secondary antibodies (Abs) used were Odyssey IRDye 680 Donkey Anti-Goat (0.5 mg); IRDye 800CW Goat anti-Rabbit IgG (LI-COR Biosciences, USA); rabbit anti-goat Alexa Fluor 488; rabbit anti-goat Alexa Fluor 647 (Molecular Probes, Invitrogen, USA). TO-PRO®-3 Iodide with far-red fluorescence from Live Technologies (Invitrogen, USA) was used as dead cell indicator.

Recombinant hGal-4 protein was purchased from BD Biosciences; LiCor blocking buffer was acquired from LI-COR Biosciences, USA; lactose was obtained from Sigma (USA) and red fluorescent cell staining CM-DiI from Vybrant, Invitrogen (USA). Control siRNA (scramble A) and for GAL4 siRNA (10 µM) was purchased from Santa Cruz Biotecknology (USA). Silencer pre-designed siRNA against Gal-4 (20 µM) and Ambion® *Silencer®* Negative Control #1 siRNA (20 µM) was purchased from Ambion (USA). Lipofectamine RNAiMax and Opti-MEM transfection reagents were obtained from Invitrogen (USA).

### Cells and Culture Conditions

Pancreatic cancer cell lines Pa-Tu-8988S (PaTu-S) and Pa-Tu-8988T (PaTu-T) were purchased from DSMZ (Germany). Other pancreatic cell lines were a kind gift from Prof. Dr. Richardson (Leiden University, The Netherlands) [32]. The cell lines AsPC1, BxPC3, MiaPaca and Panc01 were cultured in RPMI (GIBCO, Invitrogen), with 10% FCS (Lanza, Belgium) and 1∶100 Pen/Strep (GIBCO, Invitrogen) at 37°C+5% CO_2_. The cell lines Capan-I and Capan-II were cultured with 15% FCS. PaTu-S, PaTu-T and PaTu8902 were cultured in DMEM high glucose (GIBCO, Invitrogen), with 10% FCS and 1∶100 Pen/Strep at 37°C+5% CO_2_.

### cDNA Synthesis and Quantitative Real-time PCR

Total RNA was isolated from all cell lines using TriZol Reagent (Invitrogen) following the manufacturer’s guidelines. mRNA was subsequently transcribed into cDNA using the Reverse Transcription System kit (Promega, USA), as described previously [33]. cDNA from normal human pancreatic duct epithelial-like hTERT-HPNE cell line [34] was a kind gift from Dr. E. Giovannetti (Dept. of Medical Oncology, VUmc Cancer Center Amsterdam, the Netherlands). Real time (RT) PCR reactions were performed with the SYBR Green method in an ABI 7900HT sequence detection system (Applied Biosystems, USA) as described previously [35]. All oligonucleotides were designed using Primer Express 2.0 (Applied Biosystems, USA) computer software, and synthesized by Invitrogen Life Technology (USA). The reactions were carried out as follows: 2 min at 50°C, followed by 10 min at 95°C and 40 cycles of 15 sec at 95°C and 1 min at 60°C. Data are expressed as relative mRNA abundance obtained from the CT values from the target versus the endogenous reference gene *GAPDH*.

### Construction of PaTu-T Cells Expressing Gal-4

To construct PaTu-T cells that express recombinant Gal-4, the human Galectin-4 (hGal-4) gene was cloned by inserting cDNA of the hGal-4 gene in the vector pRRL-cPPT-CMV-X2-PRE-SIN-IRES-eGFP (a kind gift from Dr. A. Horrevoets, VU Medical Center, Amsterdam, the Netherlands). The entire hGal-4 open reading frame (ORF) was obtained by RT-PCR amplification, introducing NsiI/EcoRI restriction sites for insertion and a Cosac sequence before the ORF (Fwd: catatgcatcaccATGGCCTATGTCCCCGC; Rev: gaattcgatTTAGATCTGGACATAGGACAAGG). The hGal-4 insert was cloned using the EcoRI site of the vector, thus placing the hGal-4 gene under a constitutive active CMV promoter. The resulting lenti-viral construct was propagated in *Escherichia coli* BL21 (DE3) and purified by spin-column plasmid isolation kit (Qiagen, Germany). Lenti-viral production and infection of PaTu-T cells with the viral construct, resulting in the cell line PaTu-T/Gal-4, was performed as previously described [36]. A control cell line (PaTu-T/mock) was constructed by introduction of the empty vector.

### Gal-4 Knock Down (KD) in PaTu-S Cells

RNA-mediated interference was utilized to reduce Gal-4 expression in PaTu-S cells. For optimal transfection efficiency in PaTu-S cells, both Gal-4 target siRNA (40 nM final concentration) from Santa Cruz Biotechnology and silencer Pre-designed siRNA (10 nM final concentration) were simultaneously used to inhibit Gal-4 expression (PaTu-S/Gal-4-KD). A negative control (scramble A together with negative control siRNA #1) was included in the experiments (PaTu-S/mock-KD). Transfections were performed according to Invitrogen guidelines for reverse transfection in a 24-wells plate using 1 µl Lipofectamine RNAiMax and 100 µl Opti-MEM medium. To reduce Gal-4 expression in PaTu-S cells, siRNA was introduced twice with 4 days interval and the cells were transplanted into the zebrafish embryo’s 24 h after the second transfection. Gal-4 mRNA levels were measured at several time points during this experiments using quantitative RT-PCR.

### Western-Blotting

Cells were lysed at 0°C in TEA lysis buffer (Triton X-100, NaCl, MgCl, CaCl_2_, TEA pH8.2) containing protease inhibitors. Protein concentration was determined by A280 measurements and BCA determination using the Protein Assay Kit of Pierce (USA). The proteins of the cell lysates (75 µg) and culture medium (25 µl) were separated by SDS-PAGE on a 12.5% polyacrylamide gel in a discontinuous buffer system and the proteins transferred to nitrocellulose membranes (Whatman Protran, Sigma). After overnight blocking in 1∶1 LiCor blocking buffer in PBST/1% BSA (PBS with 0.05% TWEEN 20, 1% BSA), the blots were incubated for 60 min at RT with goat anti-hGal-4 (0.1 µg/ml). Mouse anti-tubulin (1∶2000 dilution) was used as loading control (cell lysates) and cell debris detection (culture medium). Secondary ABs IRDye 680 anti-Goat and IRDye 800CW anti-Rabbit IgG were used at 1∶15000 dilutions (0.07 µg/ml), in PBST/1% BSA for 1 hour at room temperature (RT) in the dark. Western-blotting analysis was performed using LI-COR Odyssey systems scanner and software.

### Cell Proliferation Assay

Cells were seeded in a 96-wells plate to approximately 1×10^4^ and 1×10^5^ cells per well and incubated overnight for cell adhesion to the plate. [^3^H]Thymidine (1 uCi/well; Amersham Biosciences, USA) was added and cells incubated for another 24 h at 37°C+5% CO_2_. Cells were harvested and [^3^H]Thymidine incorporation was assessed using a liquid scintillation MicroBeta^2^ Plate Counter 2450 (Perkin Elmer, USA).

### Immunofluorescence Microscopy

PaTu-S, PaTu-T/mock and PaTu-T/Gal-4 cells grown on glass coverslips for 24 h were washed 3 times with PBS, fixed for 30 min in 4% paraformaldehyde and permeabilized in 0.1% Triton- X-100/PBS for 5 min. Unspecific binding was blocked by quenching 10 min with PBS/glycine (0.15M) followed by 30 min with PBS/gelatine 0.2%/BSA 0.5% (PBSG). The cells were incubated with polyclonal goat anti-hGal-4 in PBSG (dilution 0.2 µg/ml) for 1 h and subsequently washed with PBS. Detection was performed after 1 h incubation at RT with 5 µg/ml of the secondary Abs (anti-goat Alexa Fluor 488 or anti-goat Alexa Fluor 647). Nuclei were stained with HOECHST (1 µg/ml in PBS) during the incubation step with secondary Ab. Actin was detected by phalloidin staining (1∶5000 in PBS, 15 min). After a final washing step in PBS and embedding using Mowiol (Kuraray Poval, Germany) several cells of each condition were visualized using a Leica M6000 B microscope with the objective lens HCX PL APO 40.0×0.85 DRY. Pictures were taken with a DFC350FXR2-095903305 Camera and analyzed using LASAF software (Leica Microsystems, Germany).

### Flow Cytometry

For detection of endogenous Gal-4, cells were first fixed in 4% paraformaldehyde for 30 min at RT, followed by cell permeabilization in PBA/0.5% saponin for 15 min at 4°C. To detect the binding of recombinant human Gal-4 (rec hGal-4) to the cell surface, procedures were performed according to Patnaik, *et al* [37]. In short, cells were harvested, centrifuged and resuspended in cold Hank’s Balanced Salt Solution (HBSS, Sigma, USA) with 500 mM lactose. Cells were subsequently collected and incubated in cold HBSS with 2% BSA for 1 hour at 4°C with gentle agitation. After this, cells were washed once with HBSS/BSA with 2 mM β-mercaptoethanol (Gibco, Invitrogen), and subsequently incubated for an hour at 4°C in HBSS/BSA/1 mM β-mercaptoethanol in the absence or presence of rec hGal-4 (5 ug/ml) to detect endogenous surface-bound Gal-4 or surface- bound rec hGal-4, respectively. To assess whether Gal-4 binding is carbohydrate dependent, the binding assays were performed in the presence of 500 mM lactose. For both surface and cytosolic analysis, cells were stained with anti-Gal-4 Ab (2 µg/ml) in PBS with 0.5% BSA and 0.02% Azide (PBA) containing 10% FCS (Sigma, USA), for 30 min at 4°C. For secondary staining, Alexa488 or Alexa647 labeled Abs were used (5 µg/ml and incubation for 30 min at 4°C). Dead cell indicator TO-PRO®-3 Iodide (1 nM) was added just previously to flow cytrometry measurements. Flow cytometry was performed using a FACScan or a FACSCalibur flow cytometer and Summit software. Dead cells defined as high TO-PRO®-3 staining were excluded from analysis.

### *In vitro* Migration Assay (“scratch”-assay)

The scratch-assay was performed as previously described by Liang et al. [38]. Cells were grown to confluency in a 24-wells plate. The cell monolayer was scraped in a straight line with a 200-µl pipette tip (Sarstedt, Germany). Photographs of the scratch were taken under an invert Leica DMI microscope at 0 h, 12 h, 24 h and 48 h for PaTu-T/Gal-4 and PaTu-T/mock cells. Photographs at each time point were taken with Leica DFC420 camera. Gap width at 0 h was set to 100%. Gap width analysis was performed with PhotoshopCS4 using the analytical ruler tool. Measurements were taken at multiple defined sites (>5) along the scratch. Each scratch was given an average of all measurements. Data are expressed as the average ± SEM of three independent experiments.

### *In vivo* Metastasis Assay

*Roy^−/−^;nacre^−/−^ casper Danio rerio* (zebrafish) were handled in compliance with local animal care regulations and standard protocols of the Netherlands. Fish were kept at 28°C in aquaria with day/night light cycles (10 h dark versus 14 h light periods). The developing embryos were kept in egg water (60 µg/ml instant ocean see salts) at 28°C before transplantation and at 35°C after transplantation.

Cancer cell transplantation was performed according to Marques *et al* [39]. Basically, cells were grown to confluency, trypsinized (GIBCO, Invitrogen) and centrifuged 5 min, at 1500 rpm. Cells were then stained in PBS containing CM-DiI at a final concentration of 4 ng/µl, for 4 min at 37°C and 15 min at 4°C. Subsequently, the cells were harvested and the cell pellets re-suspended in 100% FCS for 5 min recovery and washed twice with PBS. Finally, the cells were re-suspended in PBS for transplantation into zebrafish embryos 2 days post fertilization (dpf). Embryos were dechorionated and anesthetized with tricaine (MS-222, Sigma-Aldrich, USA). Using a manual injector (Eppendorf,Germany; Injectman NI2), the cell suspension was loaded into an injection capillary (15 µm internal- and 18 µm external-diameter). Next, approximately 100 red fluorescent cells were injected into the yolk sack of the dechorionated embryos. After injection, embryos were allowed to recover from transplantation for 1 h at RT. After this period (2 h post transplantation (hpt)), embryos were examined for the presence of fluorescent cells. Embryos containing fluorescent cells outside the transplantation area at 2 hpt were considered to be leaky and excluded from further analysis. The number of embryos alive at this stage was set as 100% for each condition independently. All other embryos were incubated at 35°C for the 3 following days.

### Imaging, Selection and Positioning of Transplanted Zebrafish Embryos

At 1, 2 and 3 days post transplantation (dpt), the embryos were anesthetized with tricaine and positioned laterally on 3% methylcellulose. Embryos were screened under a stereo DSR fluorescence Leica MZ16FA microscope. Fluorescent cancer cells outside the area of implantation were counted in every embryo. Embryos that presented more than 5 cancer cells outside the yolk were considered as being positive for metastasis and were set aside separated from the rest of the transplanted embryos. Percentage of metastasis was set as the number of embryos containing more than 5 cells outside the yolk per day relative to day zero. Total metastasis percentage is set as the total number of embryos with metastasis after 3 days relative to day zero.

### Statistics

Data are presented as mean ±SEM. Statistical analysis applied to the quantitative RT-PCR data was one-way ANOVA using Dunnett t-tests. For all other data, statistical analysis used was one-way ANOVA’s Tukey t-tests. *In vivo* metastasis assays were statistically analyzed using paired sample T-tests. Data were considered significant if *p*≤0.05.

## Results

### mRNA Expression of Gal-4 in Pancreas Adenocarcinoma Cell Lines

**Figure 1:**
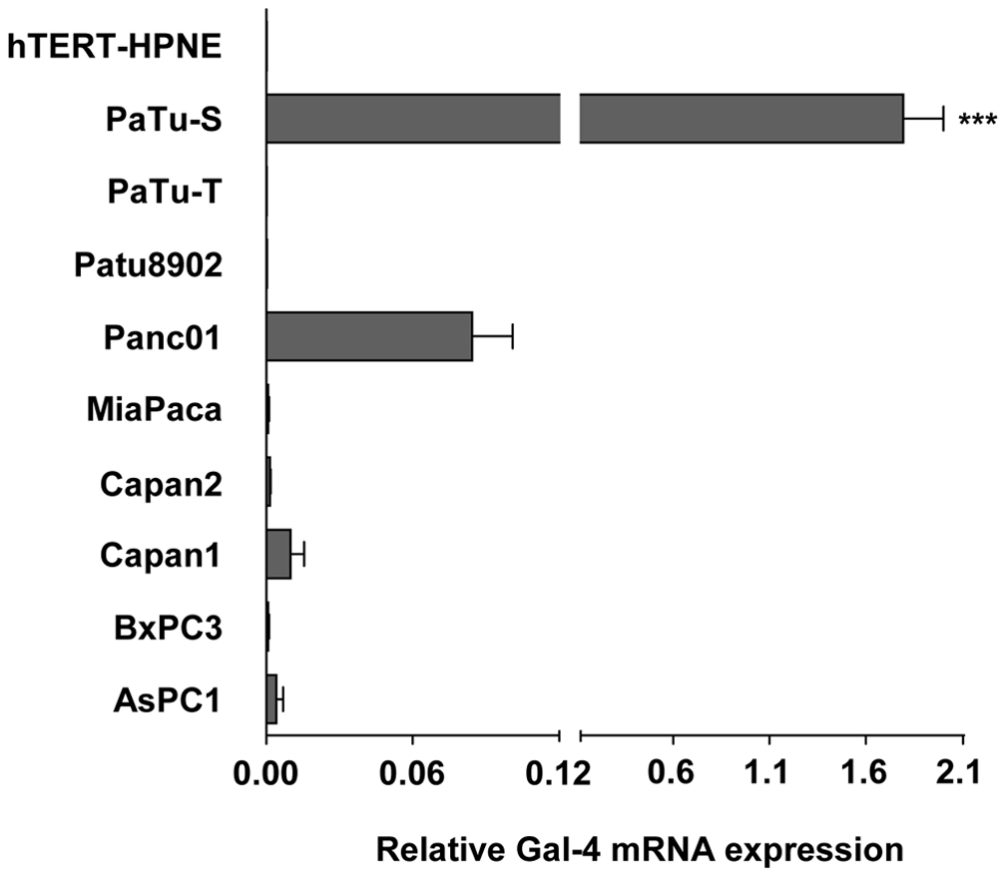
Gal-4 mRNA expression in pancreatic cancer cell lines. Gal-4 mRNA expression of normal human pancreatic duct epithelial-like cell line (hTERT-HPNE) and 9 different human pancreatic cancer cell lines was analyzed by quantitative real-time PCR and depicted as the relative amount of Gal-4 transcripts (± SEM) compared to the expression of the endogenous reference gene *GAPDH*. (*** p≤0.001 versus all other cell lines, using one way ANOVA with post Dunnett two sided t tests).

To investigate differential expression of Gal-4, mRNA levels were determined in nine different human pancreatic cancer cell lines using Real Time (RT) PCR (Figure 1). As a control for the expression in normal pancreatic duct tissue, Gal-4 mRNA levels were determined in an immortalized cell line derived from normal human epithelial pancreatic duct (hTERT-HPNE). The results demonstrated that Gal-4 mRNA levels in the normal pancreatic duct cell line were not detectable. Gal-4 mRNA levels showed a relative low abundance in eight of the nine cancer cell lines, using *GAPDH* as a household reference gene. One cell line, Pa-Tu-8988S (PaTu-S), however, showed a more than 10 times elevated expression compared to the other cell lines.

Interestingly, PaTu-S originated from the same liver metastasis of a human primary pancreatic adenocarcinoma as one of the low Gal-4 expressing cell lines, Pa-Tu-8988T (PaTu-T) [40]. These two cell lines are described to contain opposite migratory and metastatic capacities. PaTu-S and PaTu-T display a very low and a high metastatic capacity, respectively, both *in vitro* and *in vivo* using zebrafish as a model system [39]. Furthermore, it has been shown previously that most of the cell lines depicted in Figure 1 possess an increased *in vitro* migration capacity, compared to PaTu-S [32], [41]. These data led us to consider the possibility that expression of Gal-4 may restrict the migratory and/or metastatic capacity of these pancreatic cancer cells. Due to their common origin, the low migratory PaTu-S cell line and the metastatic PaTu-T cell line represent a very attractive model system to study the putative role of Gal-4 in metastasis.

### Localization of Gal-4 in PaTu-S and PaTu-T Cells

**Figure 2:**
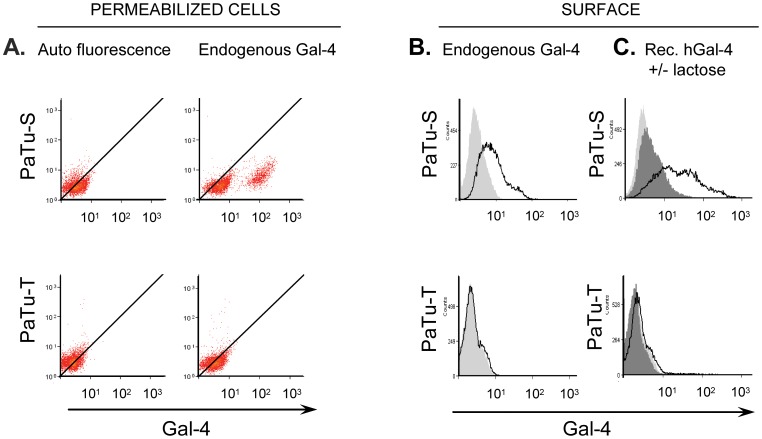
Gal-4 and Gal-4 binding sites in PaTu-S and PaTu-T cells. Detection of endogenous Gal-4, and Gal-4 ligands, in PaTu-S and PaTu-T cells by flow cytometry. A histogram of one representative experiment is depicted for each condition of least two independent experiments. **A)** Dot plots of Gal-4 staining of permeabilized PaTu-S and PaTu-T cells. Gal-4 was detected at 4°C with anti-hGal-4 Abs in fixed permeabilized cells. Secondary Abs staining without anti-hGal-4 Abs was used as background autofluorescence control. **B)** Presence of endogenous bound Gal-4 to the surface of PaTu-S and PaTu-T after washing the cells with 500 mM lactose prior to Gal-4 staining. The presence of Gal-4 was established by FACS analysis using anti-hGal-4 Abs at 4°C. Endogenous Gal-4 bound to the surface is shown by a black line. **C)** The presence of Gal-4 binding sites on PaTu-S and PaTu-T cells was determined after washing the cells with 500 mM lactose prior to Gal-4 staining. The binding of externally added recombinant (rec) hGal-4 (5 µg/ml, black line) was investigated. Binding of rec hGal-4 to the surface could be inhibited by adding lactose (dark field). Background staining with secondary Abs is depicted as light grey fields in B and C.

**Figure 3:**
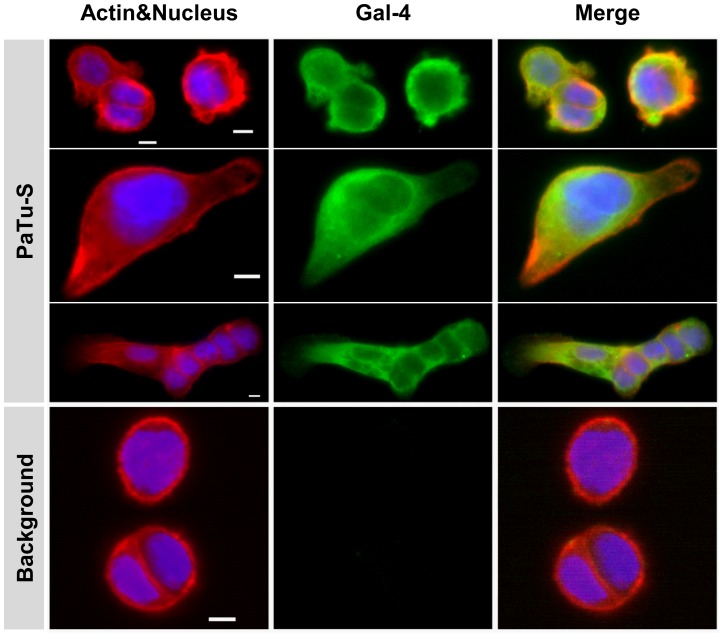
Immunocytochemical localization of Gal-4 in PaTu-S cells. Photographs of representative ICC analysis of the cellular localization of Gal-4 in PaTu-S cells. Gal-4 was detected using Alexa-labeled anti-Gal-4 Abs (green), Actin was stained using Phalloidin (red) and nucleus staining obtained using HOESCHS (blue); the third panel shows the merging of the different stainings. Bar = 25 µm.

**Figure 4:**
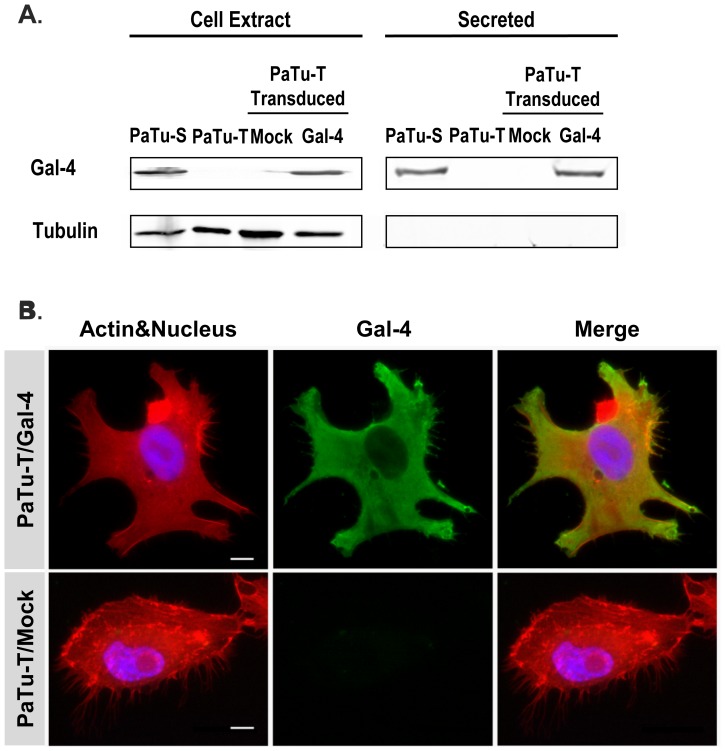
Gal-4 protein levels in PaTu-S and PaTu-T cells, and localization of Gal-4 in PaTu-T/Gal-4. **A)** Proteins from whole-cell extracts (75 ug total protein) and culture medium (4 days culture, 25 ul) of PaTu-S (P-S), PaTu-T (P-T), PaTu-T/Gal-4 (P-T/Gal-4) and PaTu-T/mock (P-T/M) were separated by SDS-PAGE. After transfer of the proteins to a nitrocellulose membrane, the blots were stained using goat anti-hGal-4 for detection of Gal-4, and mouse anti-tubulin as control for the presence of intracellular protein. **B)** Photographs of representative ICC analysis of the cellular localization of Gal-4 in PaTu-T/Gal-4 and PaTu-T/mock cells**.** Gal-4 was detected using Alexa-labeled anti-Gal-4 Abs (green), Actin was stained using Phalloidin (red) and nucleus staining obtained using HOESCHS (blue); the third panel shows the merging of the different stainings. Bar = 25 µm.

The expression and localization of Gal-4 protein in PaTu-S and PaTu-T cells was determined using flow cytometry, Immunocytochemistry (ICC) and western blotting (WB) (Figures 2–4). To determine endogenous Gal-4 of PaTu-S and PaTu-T cells, the cells were fixed and permeabilized, followed by flow cytometry using anti-Gal-4 antibodies (Abs), and fluorescent secondary Abs. The results show a clear difference in Gal-4 Ab binding between PaTu-S and PaTu-T. PaTu-S cells show a strong staining by anti-Gal-4 Abs, whereas Gal-4 staining in PaTu-T cells is hardly detectable (Figure 2A). To assess the presence of glycan ligands at the outer cell-surface that could be recognized by Gal-4, cells were first rigorously washed with 500 mM lactose to remove endogenous surface bound galectins. Subsequently, exogenous rec. hGal-4 protein (5 ug/ml) was added to the cells and Gal-4 binding was determined by flow cytometry using anti-Gal-4 Abs. The results show that still a significant level of Gal-4 staining was observed at the surface of PaTu-S cells, but not PaTu-T cells, after washing with lactose (Figure 2B), indicating the presence of strongly bound endogenous Gal-4 on the surface of PaTu-S cells. After addition of rec hGal-4 to the cells, the cell-surface Gal-4 staining strongly increased, indicating that PaTu-S cells can bind high levels of Gal-4 to their surface. By contrast, much lower levels of rec hGal-4 bound to the cell-surface of PaTu-T cells. To investigate whether the rec hGal-4 binds to the cells in a carbohydrate-dependent manner, PaTu-T and PaTu-S cells were incubated with rec hGal-4 in the presence of lactose. In the presence of 500 mM lactose the binding of hGal-4 to both cell lines was inhibited, indicating that the Gal-4 binding to the cell surface is glycan-dependent. Collectively, these results demonstrate that high Gal-4 levels are present in PaTu-S cells, whereas Gal-4 is hardly detectable in PaTu-T cells. In addition, PaTu-S cells can bind much higher levels of Gal-4 to their outer surface than PaTu-T cells, indicating that they express more Gal-4-binding carbohydrate ligands.

The cellular localization of Gal-4 was further studied using ICC. The results show high fluorescence intensity throughout the cytoplasm of PaTu-S cells (Figure 3), indicating that most Gal-4 is localized in the cytosol. High fluorescence was observed at the cytoplasmic membrane in individual cells and between cells, in particular at contact sites between neighboring cells (cell-cell contacts), whereas hardly any fluorescence was detected in the nucleus. Gal-4 is not homogenously distributed in the cytoplasm, showing some areas with higher fluorescence levels than others although there are no clear indications of specific organelles involved in accumulation of Gal-4. In PaTu-T cells, no Gal-4 could be detected using this method, establishing the flow cytometry data indicating that Gal-4 levels in PaTu-T cells are very low.

### Overexpression of Gal-4 in PaTu-T Cells

To assess a putative involvement of Gal-4 in migration, Gal-4 was overexpressed in PaTu-T cells by introduction of a viral vector containing the hGal-4 gene driven by a CMV promoter (PaTu-T/Gal-4). As a control, the viral vector without insert was transduced to PaTu-T (PaTu-T/mock). Protein expression and localization of Gal-4 in untreated PaTu-T and PaTu-S cells, PaTu-T/Gal-4 and PaTu-T/mock was analyzed by western blot (WB), ICC and flow cytometry.

To determine the level of Gal-4 expression in the cell lines, cells and medium were harvested after 4 days of culture. Proteins present in the cell extracts and medium were separated by SDS-PAGE and transferred to a nitrocellulose membrane. After staining the blots using goat anti-Gal-4 Abs, bands at 36 kDa corresponding to the apparent mass of Gal-4 were observed in PaTu-T/Gal-4 cell extract and medium, in similar amounts as observed in PaTu-S cell extract and medium, respectively (Figure 4A). As expected, PaTu-T/mock did not show detectable bands at 36 KDa. These results indicate that Gal-4 is expressed in PaTu-T/Gal-4 at similar levels compared to PaTu-S cells. Moreover, similar amounts of Gal-4 are secreted by PaTu-S and PaTu-T/Gal-4 cells.

Using ICC, we demonstrated that the intracellular distribution of Gal-4 within the PaTuT/Gal-4 cells is similar to the distribution in PaTu-S cells, whereas no Gal-4 was detected in PaTuT/mock cells (Figure 4B). The distribution of Gal-4 in these cells in the cytosol is similar to the distribution in PaTu-S cells. Gal-4 is present throughout the whole cell, except for the nucleus, similar as observed for PaTu-S. However, whereas Gal-4 is present at the plasma membrane of PaTu-S cells, hardly any Gal-4 is detected at the membrane of permeabilized PaTu-T/Gal-4 cells. In conclusion, these results indicate that PaTu-T/Gal-4 expresses and secretes Gal-4 in similar amounts as PaTu-S cells.

In addition, we determined whether endogenous, and/or added recombinant Gal-4 was bound to the cell-surface of PaTu-T/Gal-4 by flow cytometry using anti-Gal-4 Abs. Binding of anti-Gal-4 Abs to the cell surface did not differ between PaTu-T/Gal-4 and PaTu-T/Mock (data not shown). Hence, the capacity of PaTu-T/Gal-4 cells to bind Gal-4 extracellularly is unchanged compared to the untreated PaTu-T cell line.

### Overexpression of Gal-4 Diminishes the Migration Capacity of PaTu-T Cells

**Figure 5:**
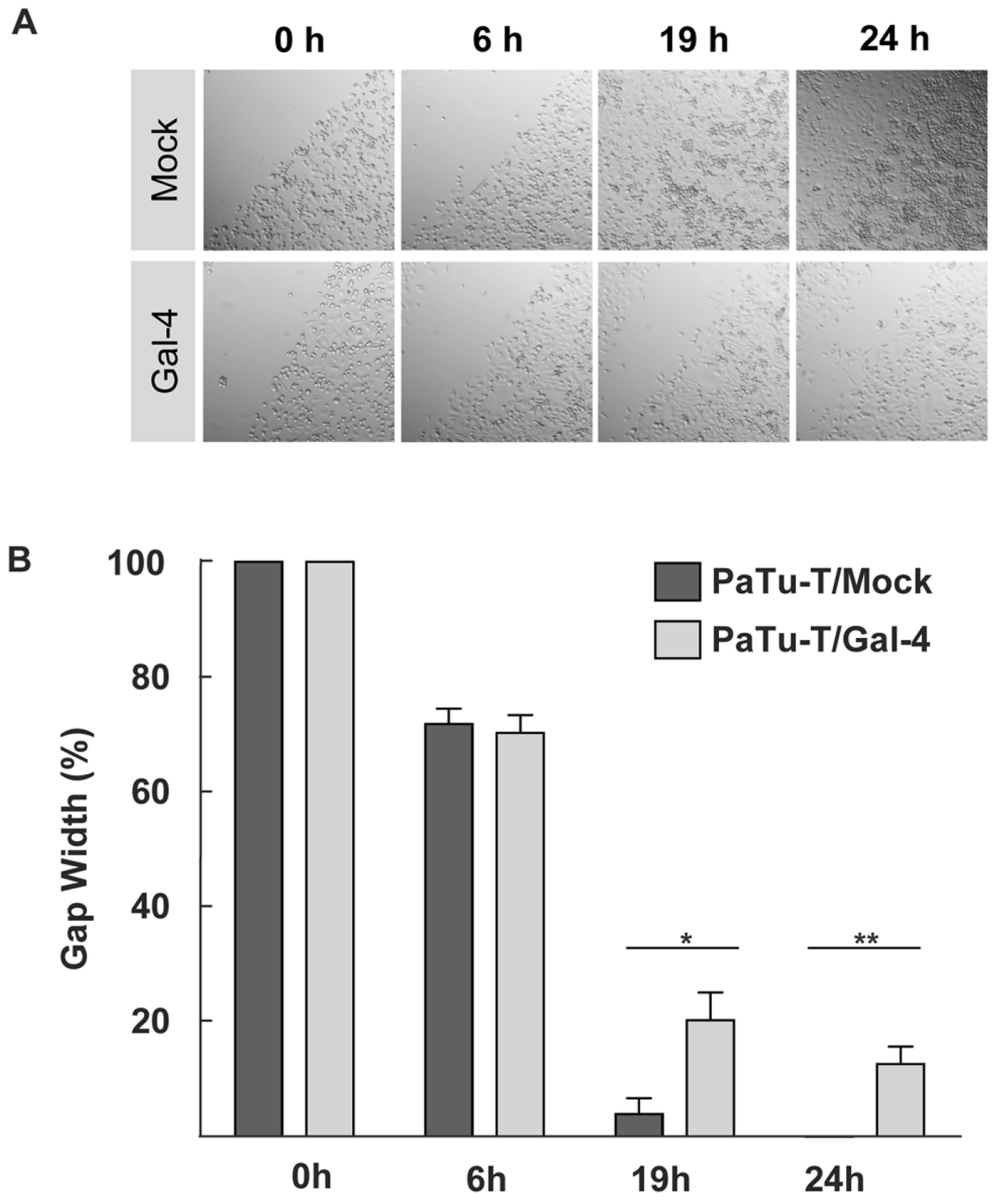
In vitro cell migration of PaTu-T cells. A scratch (wound healing) assay was performed with PaTu-T, PaTu-T/Gal-4 and PaTu-T/mock cells. PaTu-T/mock and PaTu-T/Gal-4 cells were seeded on a 24 well plate and scratched on the surface with a 200-µl pipette tip. Relative values were set at 100% of the gap width at the time of the scratch. **A)** Representative photographs at time points 0, 6, 19 and 24 hours after the wound (scratch) for all conditions are depicted. **B)** Histogram representation of data analyzed from photographs taken at 0 h; 6 h, 19 h and 24 h after the scratch. Measurements were done in duplicate in 3 separate experiments, and data are depicted as average gap width ± SEM. (* p≤0.05 and ** p≤0.01, using one way ANOVA Tukey t tests).

To examine whether Gal-4 influences the migratory behavior of PaTu-T cells, a scratch assay was performed using PaTu-T and PaTu-T/Gal-4 cells (Figure 5A–B).

PaTu-T/Gal-4 cells showed a significant decrease in migration of 16% after 19 h (*p* = 0.011) and of 13% after 24 h, compared with the mock-transduced cells. These results indicate that Gal-4 restricts or delays migration of PaTu-T cells *in vi*t*ro*. This reduction in cell migration was independent of cell proliferation as demonstrated by a proliferation assay which resulted in a similar incorporation of radioactive thymidine between mock and PaTu-T/Gal-4 cells (data not shown).

### Gal-4 Decreases Metastasis of Pancreatic Cancer Cells in Zebrafish

**Figure 6:**
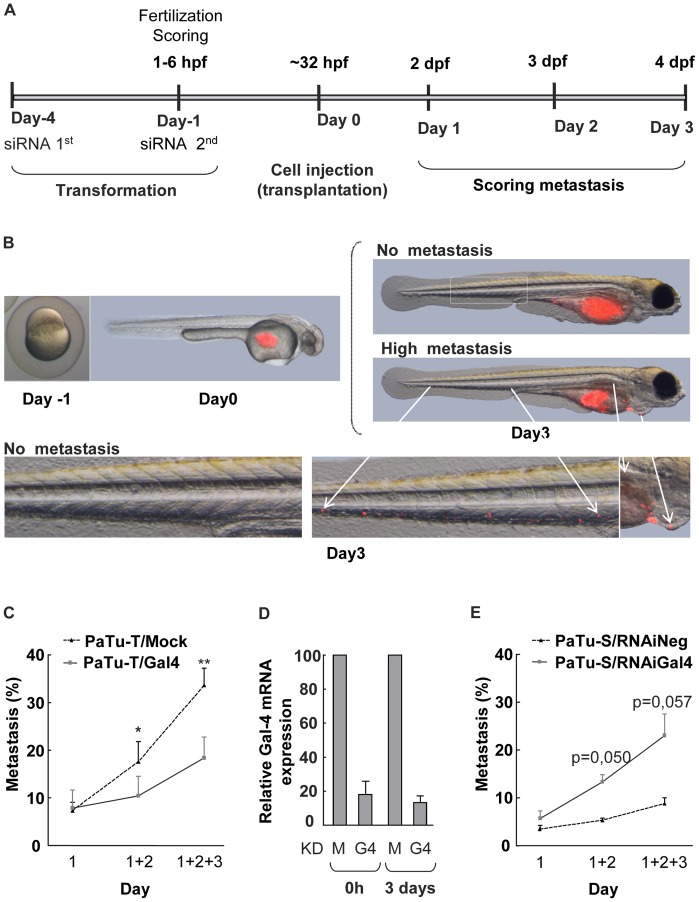
Metastasis assay of zebrafish *casper* embryos transplanted with PaTu-S and PaTu-T cells. **A)** **** Schematic depiction of the time schedule of the transplantation experiments**.** Embryos were injected at the yolk sack at 32–38 h developmental stage. siRNA was introduced twice at day -4 and day -1, respectively. At 1–6 hours post fecundation (hpf) the embryos were evaluated for viability and fitness. Scoring of metastasis formation was performed at day 1, 2 and 3 by detection of the localization of CMDiI (red) fluorescent cells. **B)** Representative photographs of a *casper* embryo at one cell developmental stage, and an embryo injected with fluorescent red cells. At day 0 cells are present only at the yolk sac of the embryo and at day 3 cells had migrated from the yolk sac throughout the embryo, including the caudal vein, hart and liver. **C)** Metastasis assay of zebrafish casper embryos transplanted with PaTu-T/Gal-4 and PaTu-T/mock cells. The number of embryos presenting metastasis is shown per day and the total percentage of embryos with metastasis formation after 3 days is depicted as a histogram. The data are derived from 4 separate experiments, and are depicted as the average metastasis formation ± SEM. **D)** Gal-4 mRNA levels of PaTu-S/Gal-4 KD (KD G4) and mock siRNA treated (KD M) PaTu-S cells were determined by quantitative RT-PCR as a control for the efficiency of the siRNA treatment. **E)** Histogram showing the total percentage of embryos, transplanted with fluorescent PaTu-S/mock-KD and PaTu-S/Gal-4-KD cells, with metastasis formation after 1–3 days. Data are derived from 3 separate experiments, and are depicted as average metastasis formation ± SEM. Significance of the data is determined by paired sample T-tests (* p≤0.05, ** p≤0.01 and, *** p≤0.001).

To assess whether expression of Gal-4 influences the metastasizing potential of pancreatic cancer cells *in vivo,* a zebrafish model was used. In this study we used *roy^−/−^;nacre^−/−^ casper* zebrafish which are fully transparent and without pigmentation until adulthood [42]. PaTu-T/mock and PaTu-T/Gal-4 were fluorescently labeled and injected in the yolk sack of embryos at 32 hpf. In parallel, PaTu-S cells were fluorescently labeled after siRNA knock down (KD) of Gal-4 and mock siRNA treatment, respectively (Figure 6A).

Directly after injection, all transplanted cells were found in the yolk sack prior to incubation at 35°C. The development of metastases was followed for 3 days dpt.

Within the first 24 hpt no differences could be observed in the onset of metastasis between the embryo groups transplanted with PaTu-T/Gal-4 versus PaTu-T/mock cells. At that time, cancer cells were found in the most posterior area of the caudal vein in 7% of the embryos (Figure 6B, C). At 2 dpt, however, 17.2% of the embryo’s transplanted with PaTu-T/mock cells showed the presence of metastasis formation, versus 9.4% of the embryos transplanted with PaTu-T/Gal-4. After 3 dpt, 32.6% of the embryos transplanted with PaTu-T/mock showed the presence of cancer cells spread throughout the whole embryo including distal parts of the embryo such as the caudal vein, head (brain and eyes) and different organs (liver, heart, intestine and gill arches). By contrast, in embryos transplanted with PaTu-T/Gal-4, cells had migrated only in 16.9% of the embryos after this 3 day period. The total metastasis formation in embryos transplanted with PaTu-T/Gal-4 after the 3 day period was significantly reduced (>15%) when compared to the embryos transplanted with mock transduced PaTu-T cells.

The treatment of PaTu-S cells with siRNA (PaTu-S/Gal-4-KD) resulted in a reduction of Gal-4 mRNA expression by >80% (Figure 6D). Zebrafish embryos were transplanted with PaTu-S/Gal-4-KD and PaTu-S cells treated with mock siRNA (PaTu-S/Gal-4-mock), and metastasis assays were conducted as described above. Metastasis formation in the PaTu-S/Gal-4-KD transplanted cells versus the control embryo group was very similar after 1 dpt, however a significant increase in metastasis of the Gal-4 KD transplanted group was observed after 2 dpt, where 13.3% of the embryos displayed migrating cells, versus 5.3% in the control group. Within 3 days, a total of 23.0% of the embryos demonstrated PaTu-S/Gal-4 KD cells spread throughout the embryos in particular within the caudal vein. Thus, using these cell lines an increase of 14% in total metastasis formation of PaTu-S/Gal-4-KD cells was observed, compared to the mock-treated cells after the 3 day period (Figure 6E). Collectively, these results indicate that the presence of Gal-4 reduces metastasis formation in the pancreatic cell lines PaTu-T and PaTu-S *in vivo* within 2 and 3 dpt.

## Discussion

Gal-4 is highly expressed in the healthy intestinal tract, where it may act as an innate defense lectin by killing human blood group antigen-expressing bacteria through its galactoside-binding activity [43]. In colon carcinoma, however, reduced levels of Gal-4 are observed compared to the normal colon crypt epithelia [44], [45]. In this study we evaluated Gal-4 mRNA levels in a panel of pancreatic cancer cell lines, and found that Gal-4 expression levels were similar or slightly elevated compared to immortalized normal human pancreatic duct epithelial-like cells in 8 of the 9 cancer cell lines tested. Only in the pancreatic tumor cell line PaTu-S, Gal-4 is highly expressed at both mRNA and protein level. These findings do not seem to be in accordance with several studies that indicate a high expression of Gal-4 in pancreatic adenocarcinoma tissue, and other tumors of tissues where Gal-4 is not normally expressed, such as liver, breast, and ovary [24], [28], [30], [46], [47], [48], [49].

To increase our understanding of the putative role(s) of Gal-4 in cancer, we determined its expression and function in two pancreatic cell “sister” cell lines which originate from the same liver metastasis of a patient with pancreatic adenocarcinoma [40]. We demonstrated that these cell lines, PaTu-S and PaTu-T, show high and low Gal-4 expression, respectively. Interestingly, the cell lines show also many other opposite characteristics, the major being that PaTu-S cells show E-cadherin expression, maintain cell-cell contacts and have low migratory properties, whereas PaTu-T cells have lost E-cadherin expression and are highly metastatic both *in vitro*, and in an *in vivo* zebrafish model [39]. Thus, in addition to other factors, these cells show an inverse association between Gal-4 expression and tumor progression. This is in agreement with studies in other types of cancer that indicate that high Gal-4 levels are found in particular in cancer cells that maintain cell-cell contacts, and show a lowered expression in more advanced stages of the disease [25], [26], [44], [49], [50].

Our results are novel in demonstrating that Gal-4 expression inhibits migration and metastasis of pancreatic cancer cell lines both *in vitro* and *in vivo* using zebrafish as experimental model. Overexpression of Gal-4 in PaTu-T cells significantly delays metastasis of these cells. In addition, lowering expression of Gal-4 in PaTu-S cells by siRNA increases the metastatic properties of PaTu-S.

The use of the zebrafish (*Danio rerio*) embryo model for studying the migration and invasion of cancer cells is rapidly increasing due its versatility and reliability (reviewed in [51]). Whereas most studies in zebrafish have been performed with different cancer cell lines, also a few xenotransplantation studies using primary cancer cells have been reported. These latter studies include the transplantation of small pieces or dissociated cells from human chronic pancreatitis tissue, and of carcinoma tissue from human pancreas, colon and stomach in zebrafish embryo’s and could clearly establish migration and metastasis of pancreatic adenocarcinoma cells from the tumor tissue, while the chronic pancreatitis cells were non-invasive [32], [39]. Two other studies used engraftment of human leukemia primary cells and showed that these were able to proliferate and migrate throughout the fish [52], [53]. Furthermore, high interspecies conservation of molecular pathways has been shown between zebrafish and mammals ([54], [55], [56] and reviewed in Davidson, A.J. and Zon, L.I. 2004). Clearly, the zebrafish model is an attractive model system that does overcome some of the major drawbacks of using mammals as a xenograph model such as immuno-permissiveness, long duration time for human cell engraftment to become visible, single cell imaging and high costs [51].

The mechanism by which Gal-4 inhibits migratory properties of the cells is still unclear. The observation that down regulation of Gal-4 in PaTu-S cells resulted in enhanced migratory properties of the cells may be explained by loss of Gal-4 as an external adhesion molecule that stabilizes cell-cell contacts [57], [58], [59]. Our data indicate that PaTu-S cells secrete and bind Gal-4 to their outer surface. The binding of Gal-4 to the PaTu-S outer surface, as demonstrated by fluorescent cell cytometry using anti-Gal-4 Abs, is glycan-dependent since most surface-bound Gal-4 could be displaced by adding lactose. In addition, using immunofluorescence microscopic analysis, Gal-4 was detected at the cytoplasmic membrane in particular at cell-cell contact sites, indicating a role in cell-cell adhesion. Such Gal-4 localization resembles those described in studies of Gal-4 localization in colon adenocarcinoma cells [60]. Down regulation of Gal-4 expression may diminish adhesion of the tumor cells to each other, and thus may facilitate escape of the cancer cells from the tumor site. Alternatively, or in addition, the presence of cytosolic Gal-4 may enhance survival of tumor cells in a nutrient depleted environment, as proposed by Huflejt and Leffler [46].

Our data showed that artificial expression of Gal-4 in PaTu-T cells inhibits migration of the cells both *in vitro* and *in vivo*. Remarkably, we could detect Gal-4 in the cytosol of PaTu-T/Gal-4 cells but we observed hardly any Gal-4 binding at their surface. Despite the fact that these cells express and secrete similar levels of Gal-4 as compared to PaTu-S cells, Gal-4 can bind only to a very limited extent to the outer cell-surface of PaTu-T cells. Apparently, PaTu-S and PaTu-T cells differ in their cell surface glycosylation, resulting in different levels of glycan moieties that can act as ligands for Gal-4 in these cell lines. This indicates that the inhibitory role of Gal-4 in the migratory properties of PaTu-T/Gal-4 cells is not likely due to a role of Gal-4 as a surface adhesion molecule. Thus, collectively our data suggest that the inhibition of metastasis observed in PaTu-T/Gal-4 is mainly due to a cytosolic role of Gal-4. It has been described that cytosolic Gal-4 can be involved in apical membrane glycoprotein trafficking [9], [61]. In PaTu-T cells, low levels of Gal-4 may limit membrane transport of its own ligands. However, we could not detect an enhanced capacity of Gal-4 binding by PaTu-T cells after overexpression of Gal-4, making this possibility unlikely. Alternatively, it may be possible that Gal-4 contributes to inhibition of metastasis by down regulation of Wnt signaling target genes as has been shown for colon rectal cancer (CRC) [50]. The latter study demonstrates that Gal-4 negatively regulates cell cycle, cell proliferation, migration and motility of colorectal cancer cells, and the authors proposed that Gal-4 acts as a tumor suppressor in CRC [50].

In conclusion, this study is the first to demonstrate a direct effect of Gal-4 in the metastatic properties of pancreatic cancer cells. This effect may be dependent on multiple functions of Gal-4, and it is likely that Gal-4 expression is finely regulated in the initial stages of tumor formation. Gal-4 may be upregulated early in pancreatic cancer, thereby acting as an “adhesin” at the cell-surface, as well as a cytosolic inhibitor of migratory properties. Later in tumor progression Gal-4 and its cellular glycan ligands may be down regulated, thereby facilitating escape from the tumor site by loss of cell-cell interaction, and enhanced migratory properties. A similar Gal-4 expression pattern has been observed in human ileal carcinoid tumors, where expression of Gal-4 is generally higher in primary rectal carcinoid compared with metastatic tumors [26], [44]. Increased understanding of the significance of Gal-4 in the metastatic process will increase our insight in the abnormal expression of galectins and possibly other carbohydrate binding proteins, in relation to their interaction with self- or tumor glycan antigens and cancer progression.
